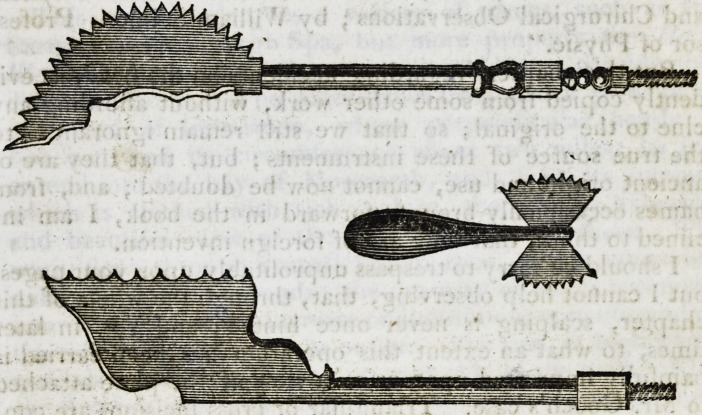# Coincidence in the Figure of Instruments Proposed in the Seventeenth Century and Those of Mr. Hey

**Published:** 1817-04

**Authors:** Samuel Spencer


THE LONDON

### Medical and Physical Journal.

4 OF VOL. XXXVII.] APRIL, 1817. [NO. 218.

"For many fortunate discoveries in medicine, and for the detection of nume-
"rous errors, the world is indebted to the rapid circulation of Monthly
"Journals; and there never existed any work to which the Faculty in
"EUROPE and AMERICA were under deeper obligations than to the
"Medical and Physical Journal of London, now forming a long, but an
"invaluable, series."—RUSH.

*For the London Medical and Physical Journal.*

### *Coincidence in the Figure of Instruments proposed in the Seventeenth Century and those of Mr. Hey;* by SAMUEL SPENCER, Esq.

IN turning over the pages of an old book to beguile the hours under the pressure of a deep affliction, I met with the saws, as above sketched. Their resemblance to what amongst us are very properly called Hey's Saws, interested me in their further history, and induced me to turn to the chapter on Fractures of the Skull, in the same work; and I there found them spoken of under the appellation of Head-Saws. No one will suppose, that the ancients used them with a view of saving any portion of the cranium, the merit of that recommendation rests entirely with Mr. Hey and Dr. Cockel; but, as a matter of curiosity connected with the obscure history of these important instruments, I fancied that a brief mention of them, with the above sketch, might not be altogether uninteresting to some, at least, of your numerous readers.

The chapter alluded to is divided into many sections, and the section that first notices these instruments begins thus—

"What must be done, when fractured or depressed on one side only?"

"In this case, you must use small head-saws to divide the scull, and cut off, without compression, so much of the bone as shall be needful, without danger of pressing down the broken part of the bone upon the dura-mater, from whence a great deal of mischief might ensue; by this means, you raise the depressed side, and make way to vent sanies and ichorous matter." And, in another place, speaking of them singly, "It cuts away the distances between the holes made in the skull with the trepan, as also fissures that do not penetrate, and scrapes away the rottenness of the cranium; all which it does without fear or danger."

The book from which I have taken the above was published in London, 1685, under the title of "Select Physical and Chirurgical Observations; by William Salmon, Professor of Physic."

But this chapter, regarding accidents on the head, is evidently copied from some other work, without affording any clue to the original; so that we still remain ignorant as to the true source of these instruments; but, that they are of ancient origin and use, cannot now be doubted; and, from names occasionally brought forward in the book, I am inclined to think, that they are of foreign invention.

I should be sorry to trespass unprofitably upon your pages, but I cannot help observing, that, through the whole of this chapter, scalping is never once hinted; and yet, in later times, to what an extent this operation has been carried is painfully impressed upon us on a view of the plate attached to Mr. Gooch's case. Triangular or cross incisions are constantly spoken of, and these not to be used hastily or indiscriminately; for they frequently, it is observed, "only make work where there is none, or would be very little. Except in cruel accidents, abstain from all manner of manual operations, save shaving off the hair." And, in speaking of the trepan: "It is not to be used too hastily, for many dangerous fractures are daily seen to be cured without it; there is not one in ten that truly requires it." These remarks, guarding us against too hasty proceedings in accidents happening to the head, come pretty close to the approved maxims of modern surgery; and, in the interval from their first promulgation up to our own day, it would have been well for many a sufferer if they had never been lost sight of; happily, happily, this part of surgery now rests upon a steady basis, chiefly, if not entirely, through the discerning mind of an Abernethy, and the splendid talents of Mr. John Bell.

*Duffield, near Derby;*

*Jan.* 10, 1817.

---

*For the London Medical and Physical Journal.*

## Figures and Tables

**Figure f1:**